# The regulatory mechanism of neutrophil extracellular traps in cancer biological behavior

**DOI:** 10.1186/s13578-021-00708-z

**Published:** 2021-11-10

**Authors:** Hui Wang, Yiyin Zhang, Qianling Wang, Xiaoli Wei, Hua Wang, Kangsheng Gu

**Affiliations:** Department of Oncology, The First Affiliated Hospital of Anhui Medical University, No. 218 Jixi Road, Hefei, 230022 Anhui People’s Republic of China

**Keywords:** Neutrophil extracellular traps, Cancer, Tumor microenvironment, Signal pathway

## Abstract

As the predominant host defense against pathogens, neutrophil extracellular traps (NETs) have attracted increasing attention due to their vital roles in infectious inflammation in the past few years. Interestingly, NETs also play important roles in noninfectious conditions, such as rheumatism and cancer. The process of NETs formation can be regulated and the form of cell death accompanied by the formation of NETs is regarded as “NETosis”. A large amount of evidence has confirmed that many stimuli can facilitate the release of NETs from neutrophils. Furthermore, it has been illustrated that NETs promote tumor growth and progression via many molecular pathways. Meanwhile, NETs also can promote metastasis in many kinds of cancers based on multiple studies. In addition, some researchs have found that NETs can promote coagulation and cancer-associated thrombosis. In the present review, it will highlight how NETosis, which is stimulated by various stimuli and signaling pathways, affects cancer biological behaviors via NETs. Given their crucial roles in cancer, NETs will become possible therapeutic targets for inhibiting proliferation, metastasis and thrombosis in cancer patients.

## Introduction

Neutrophil extracellular traps (NETs) are net-like structures composed of granule proteins and nuclear components (such as DNA and histones) [1]. DNA is decorated with granule proteins, including neutrophil elastase (NE), myeloperoxidase (MPO), matrix metalloproteinase 9 (MMP-9) [1], calprotectin [2], cathepsin G (CG) and proteinase 3 (PR3) [3]. Furthermore, mitochondria can also serve as a source of DNA for NET formation [4]. NETs were first recognized as a novel host defense mechanism. NETs have been shown to trap diverse pathogens, including bacteria [1], fungi [5, 6], viruses [7] and protozoan parasites [8]. Apart from infectious inflammation, NETs have been found to be involved in cancer [9] and sterile inflammatory diseases, such as ventilator-induced lung injury [10], lupus nephritis [11] and acute pancreatitis [12]. Furthermore, NETs also play a significant role in atherosclerosis disease [13]. A study has confirmed that deficiency of EGF-like repeats and discoidin I-like domain 3 can improve adverse cardiac healing through polarization of pro-inflammatory macrophage which is mediated by NETs [14]. In particular, NETs are even associated with preeclampsia and central nervous system diseases [15, 16].

NETs are released into the extracellular environment in response to relevant stimuli. The formation of NETs involves a unique form of cell death that is dependent on the generation of reactive oxygen species (ROS) by NADPH oxidase, which is different from apoptosis and necrosis [17]. Steinberg and Grinstein regarded this form of cell death accompanied by the formation of NETs as “NETosis” [18]. When neutrophils encounter relevant stimuli, they undergo morphological changes. The most salient morphological differences seen in cells undergoing NETosis but not apoptotic and necrotic cells are disintegration of the nuclear envelope and mixing of nuclear and cytoplasmic material, loss of internal membranes, and disappearance of cytoplasmic organelles. Cell death is initiated by ROS [17]. Decondensation of chromatin is a critical event for NETosis. NE exits azurophilic granules and translocates to the nucleus. Then, MPO translocates to the nucleus. MPO cooperates with NE to induce nuclear decondensation [19]. Peptidylarginine deiminase 4 (PAD4), which can promote citrullination of histones, is highly expressed in neutrophils. The hypercitrullination of histones by PAD4 promotes chromatin decondensation [20]. Next, the cell membrane breaks, and NETs are released [17]. Various stimuli have been shown to contribute the production of NETs, then inhibitors of NADPH oxidase and NE prevent NET formation [17, 19]. Therefore, investigating the signaling pathways that regulate the formation of NETs and identifying specific inhibitors may provide possibility for the treatments of human cancers.

Due to its vital roles in infectious and sterile inflammation, NETs have attracted increasing attention in the last two decades [1, 10]. In the area of malignancy, NETs have been shown to promote thrombosis, proliferation and metastasis of cancer cells [21], cause organ damage in cancer patients [9] and even predict the prognosis of cancer patients [22, 23]. In this review, we will highlight the mechanisms by which NETosis, which is stimulated by various stimuli and signaling pathways, promotes tumor progression and metastasis and cancer-associated thrombosis via NETs.

## Molecular pathway of NETosis stimulated by various agonists

**Fig. 1:**
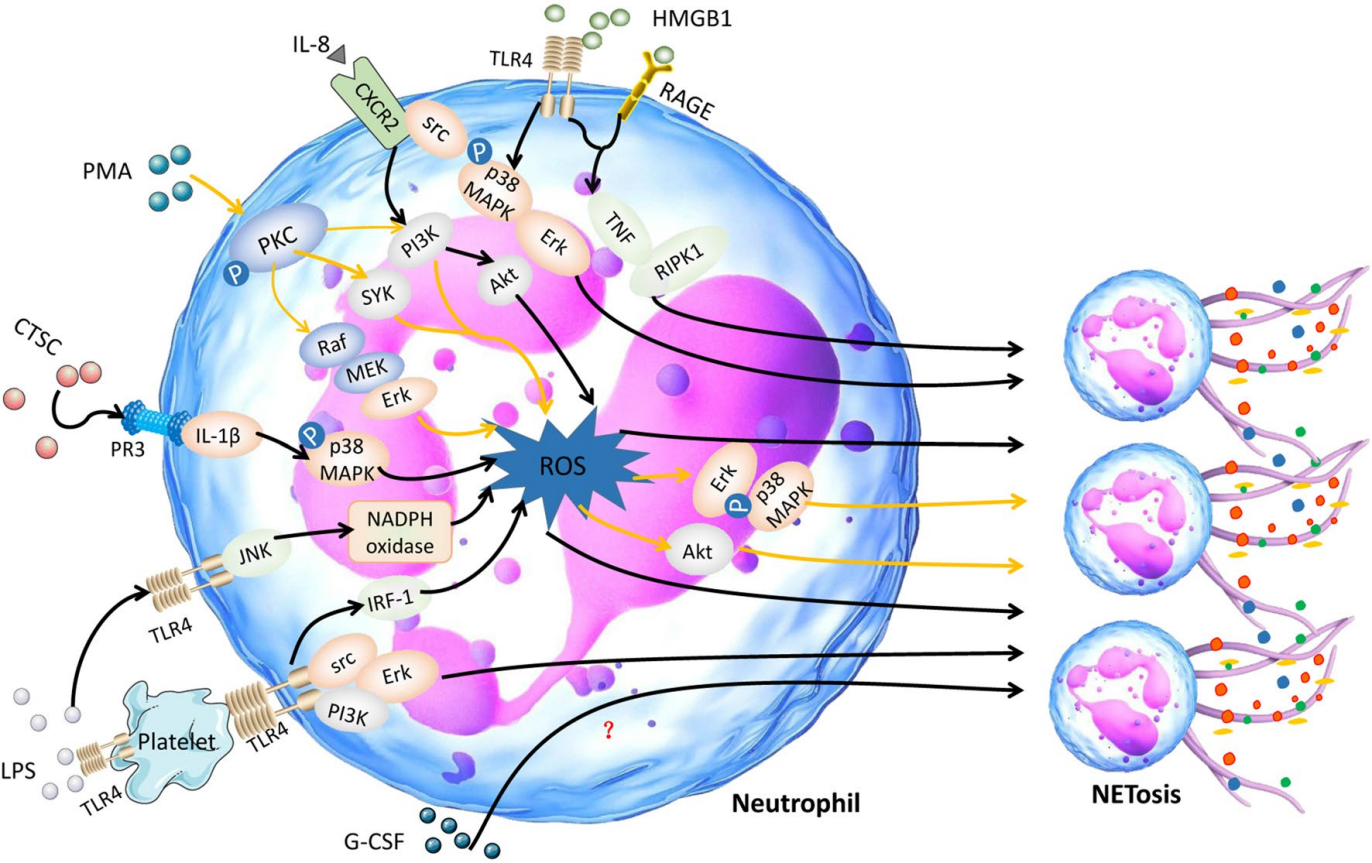
Molecular pathway of NETosis stimulated by various agonists. Many agonists which can induce the formation of NETs are described, including LPS, PMA, HMGB1, G-CSF, IL-8 and CTSC

To date, many stimuli have been confirmed to induce the release NETs from neutrophils, such as lipopolysaccharide (LPS) [24] and phorbol 12-myristate 13-acetate (PMA) [1]. Some cytokines and proteins, including high mobility group 1 (HMGB1) protein [25], cathepsin C (CTSC) [26], granulocyte colony stimulating factor (G-CSF) [27] and interleukin-8 (IL-8) [28], can also stimulate the formation of NETs. A summary of the molecular pathways by which NETosis is activated by various agonists is shown in Fig. 1.

### IL-8

IL-8, which is also named C-X-C motif chemokine ligand 8 (CXCL8), is a member of the CXC subfamily of chemokines. IL-8 is released by not only malignant cells but also stromal cells in the tumor microenvironment [29]. The plasma levels of IL-8 are higher in cancer patients than in healthy individuals [30]. IL-8 is increased in breast cancer tissues compared to adjacent normal breast tissues [31]. IL-8 secreted from tumors promotes the release of NETs [32, 33]. Furthermore, IL-8 is positively correlated with NETs in non-small cell lung cancer (NSCLC) and melanoma [32]. IL-8 can easily bind with C-X-C motif chemokine receptor 1 (CXCR1) and C-X-C motif chemokine receptor 2 (CXCR2) [34]. Agonists of CXCR1 and CXCR2 are the main mediators of NETosis induced by cancer. Blocking CXCR1 and CXCR2 in mice with breast cancer with reparixin, a specific small-molecule inhibitor of CXCR1 and CXCR2, results in decreased levels of NETs [35, 36]. Podaza et al. [37] found that plasma from chronic lymphocytic leukemia patients could promote the formation of NETs through the plasmatic IL-8-CXCR2 axis. In diffuse large B cell lymphoma (DLBCL), the IL-8-CXCR2 axis induces NETosis via src, p38 and extracellular-signal-regulated kinase (ERK) rather than the phosphoinositide 3-kinase (PI3K) signaling pathway [38]. However, in another study, IL-8 was found to promote the phosphorylation of serine/threonine-protein kinase (AKT), a key mediator downstream of the PI3K signaling pathway. The IL-8-CXCR2 axis mediates NET formation via the PI3K/AKT/ROS axis in tumor-infiltrating neutrophils [39]. In our ongoing study, we also have found that IL-8 can influence the biological behavior of gastric cancer (GC) by promoting the release of NETs through CXCR1/2. These studies demonstrate formation of NETs regulated by IL-8 and its receptors, CXCR1 and CXCR2, via various signaling pathways.

### G-CSF

G-CSF, a cytokine produced by leukocytes, endothelium, and tumors, is increased in the peripheral blood of cancer patients [40–42]. Some studies have shown increased generation of NETs in tumors induced by G-CSF [43, 44]. G-CSF can directly stimulate neutrophils to release NETs in cancer patients [45]. However, the molecular pathway by which G-CSF promotes NETosis has not been clearly identified.

### PMA

PMA (also named TPA), a potent neutrophil activator, results in morphological changes in neutrophils that are quite different from those typical of apoptosis or necrosis [46, 47]. Some studies have confirmed that PMA can promote the release of NETs from neutrophils in both healthy donors and cancer patients [48–50]. Ermert et al. [51] found that PMA can promote the generation of ROS in neutrophils in a mouse model, and neutrophils from NADPH oxidase-deficient mice failed to produce ROS and did not die or release NETs upon stimulation. In chronic granulomatous disease patients, the neutrophils have NADPH oxidase mutations and are thus unable to generate ROS even upon PMA activation [17]. PMA promotes the formation of NETs by NADPH oxidase-mediated ROS generation. Furthermore, the nonphysiological agent PMA is an activator of protein kinase C (PKC) [52]. Hakkim et al. confirmed that the Raf-MAP kinase ERK kinase (MEK)-ERK pathway is upstream of NADPH oxidase and downstream of PKC. Blocking PKC with staurosporine, a PKC inhibitor, results in decreased formation of NETs. PMA stimulates the generation of NETs through the Raf-MEK-ERK signaling pathway via the activation of PKC [53]. However, in another study, ERK was found to be downstream of ROS generation and upstream of P38 mitogen-activated protein kinase (MAPK) signaling during PMA-induced NETosis [54]. Moreover, activation of Akt is dependent on NADPH oxidase-mediated ROS production, which was essential for the NETosis induced by PMA [55]. Spleen associated tyrosine kinase (Syk) and PI3K, which mediate the generation of ROS, are crucial for NETosis elicited by PMA [56, 57].

### LPS

LPS, a main component of the gram-negative bacteria cell wall, has been identified as a potent stimulator of the formation of NETs [58, 59]. LPS can stimulate NETosis in tumors [60, 61]. Furthermore, LPS can upregulate NADPH oxidase in neutrophils [62]. Khan et al. [63] found that LPS induces activation of c-jun N-terminal kinase (JNK), which is upstream of NADPH oxidase, in a toll-like receptor 4 (TLR4)-dependent manner in neutrophils. Both JNK activation and TLR4 signaling are important for LPS-mediated ROS production and NETosis in neutrophils. However, LPS does not induce NET formation in purified neutrophils [64], which is consistent with another study reporting that LPS indirectly promotes NET generation [65]. Some research has shown that LPS-induced NETosis depends on the presence of platelets [66, 67]. The structure of LPS might result in these two different consequences [68]. LPS promotes the activation of platelets through platelet TLR4, inducing the interaction of platelets with neutrophils and the generation of NETs [69, 70]. NETosis induced by LPS-stimulated platelets is dependent on ROS. This process is mediated by interferon regulatory factor 1 (IRF-1) [71]. However, in another study, ROS were found to not be involved in the NETosis induced by platelets, which was instead activated by LPS and platelet-triggered NET release through the ERK, PI3K, and Src kinases [67]. It shows that PKC and generation of ROS are essential for PMA induced NETosis.

### HMGB1

HMGB1 protein is both a nuclear factor and a secreted protein [72]. HMGB1 is released by living immune cells or passively released from dead, dying, and injured cells [73]. Several studies have demonstrated that HMGB1 is prone to binding with receptor for advanced glycation end products (RAGE), TLR2, TLR4 and TLR9 [74–76]. Tadie et al. found a role of HMGB1 in contributing to NET formation, and HMGB1 was found to induce the generation of NETs both in vitro and in vivo through a TLR4-dependent mechanism [25, 77]. This result is consistent with a study conducted by Zhou and colleagues in lung cancer. The researchers found that the downstream molecules of TLR4, p38 MAPK and ERK, were activated during the formation of NETs [78]. Moreover, HMGB1 was found to promote NET formation via tumor necrosis factor (TNF) and receptor-interacting-protein kinase-1 (RIPK1) kinase activity during tumorigenesis of skin [79].

### CTSC

CTSC, also known as dipeptidyl peptidase I, is a lysosomal cysteine protease essential for many serine proteases, including CG, NE, PR3, granzymes A/B and mast cell chymases [80–82]. A study discovered that CTSC secreted by tumors promotes the formation of NETs in breast cancer cells. CTSC induces neutrophil ROS production and the formation of NETs by activating the neutrophil membrane-bound PR3-interleukin-1β (IL-1β)-p38 axis [83].

## NETs and cancer

Neutrophils play an important role in cancer. Tumor-associated neutrophils are divided into two phenotypes according to their functions in the tumor microenvironment: the antitumor N1 versus the protumor N2 phenotype [84]. In a spontaneous intestinal tumorigenesis model, low-density neutrophils display clear features of N2 neutrophils and spontaneously undergo NETosis via complement 3a receptor signaling [85]. Several studies have confirmed the presence of increased levels of NETs in advanced cancer patients, including DLBCL and esophageal and lung adenocarcinoma patients [22, 38, 86]. NETs can also accelerate deterioration of colorectal cancer (CRC) [87]. Moreover, in our two previous studies, we have discovered that NETs and its key component MPO are prognostic factors affecting poor survival in patients with GC [22, 88]. As a serum biomarker, NETs have a better diagnostic value than carcinoembryonic antigen and carbohydrate antigen 19-9 in GC. The level of NETs is inversely correlated with short-term efficacy in GC patients who have received medical treatment [22]. NETs are also associated with tumor burden and have been shown to promote growth, progression and metastasis in cancer [89–91]. Furthermore, a relationship between NETs and cancer-associated thrombosis has been confirmed [27]. The next three parts will highlight the mechanisms of NETs in tumor growth, progression, metastasis and cancer-associated thrombosis.

### NETs promote tumor growth and progression

**Fig. 2:**
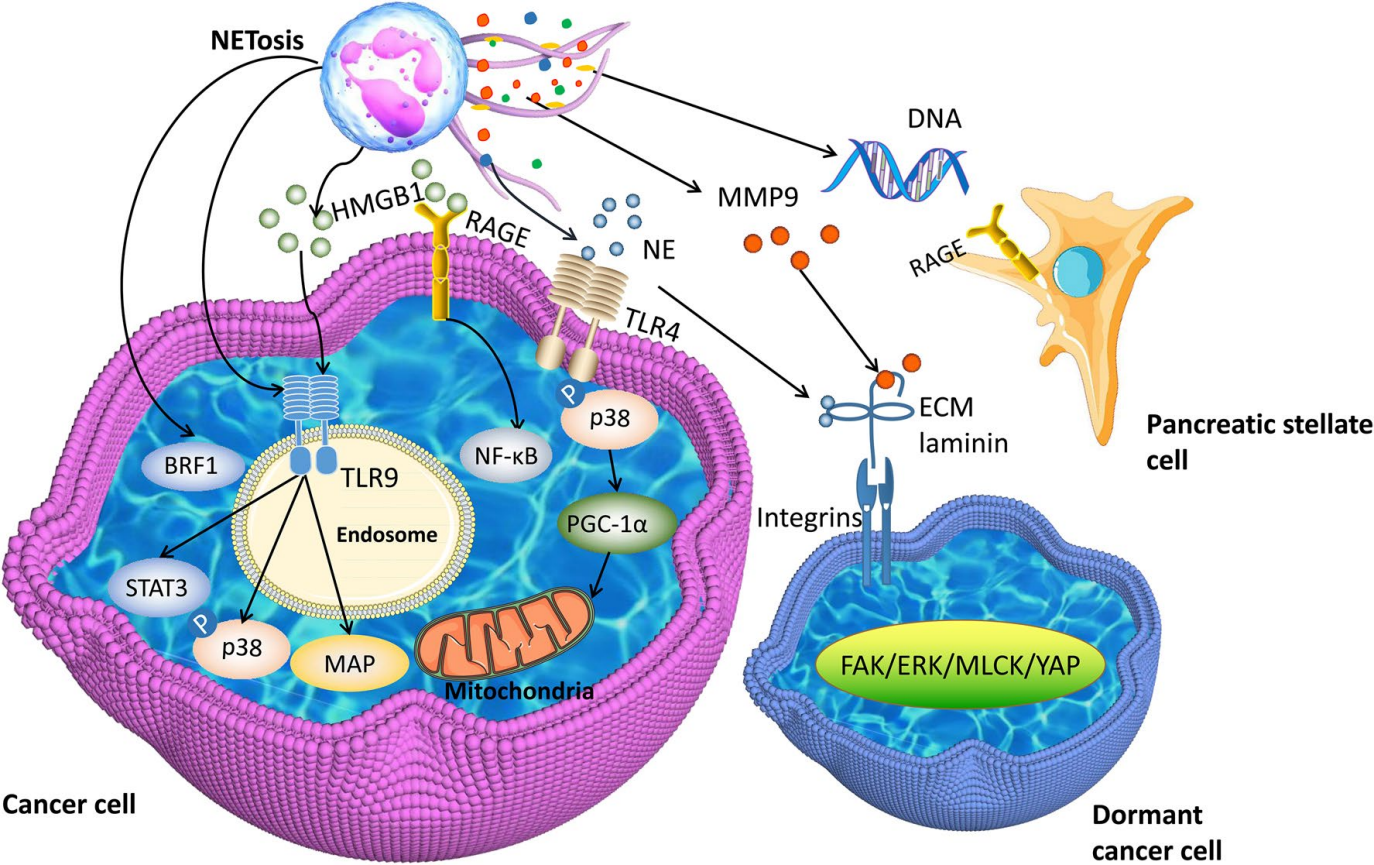
NETs promote tumor growth and progression via many molecular pathways. HMGB1, released by NETs, can promote tumor growth by binding with TLR9 or RAGE to activate STAT3, p38, MAP and NF-κB. NE directly alters metabolism and upregulates mitochondrial biogenesis in colon cancer cells via the TLR4-PGC1-α pathway and thus promotes tumor growth. NETs awaken cancer cells and cleave laminin via NE and MMP-9 through the activity of integrin α3β1 and FAK/ERK/MLCK/YAP signaling. NET DNA drives pancreatic tumor growth by activating pancreatic stellate cells through interaction with RAGE. In our ongoing study, we have illustrated that NETs can promote tumor growth by BRF1 in GC cells

NETs have been shown to promote tumor growth and progression. In a nonalcoholic steatohepatitis (NASH) model, the progression of NASH to hepatocellular carcinoma (HCC) can be reduced by blocking NETs [92]. In glioma, NETs promote the proliferation of cancer cells [39]. To date, some roles of NETs in tumor growth and progression have been identified (Fig. 2).

NETs can directly increase TLR9 expression in DLBCL and promote tumor progression via the NF-κB, signal transducer and activator of transcription 3 (STAT3) and p38 pathways [38]. Furthermore, the mechanisms by which NETs promote tumor growth and progression have been confirmed.

HMGB1, a damage-associated molecular pattern, has been shown to participate in the growth and progression of cancer [93, 94]. HMGB1 is a constituent protein of NETs [21, 95]. In glioblastoma, HMGB1 derived from NETs promotes the proliferation of cancer cells by interacting with RAGE and activating the NF-κB pathway [39]. In another experiment performed by Tohme et al. [21], HMGB1 released from NETs interacted with TLR9 in MC38 CRC cells and then activated the MAP kinase pathway to exert a protumorigenic function.

DNA is a significant element in NETs. Miller-Ocuin et al. found that DNA released from NETs promotes the proliferation of Panc02 murine pancreatic cancer cells. NET DNA drives pancreatic tumor growth in a murine subcutaneous tumor model by activating pancreatic stellate cells through interaction with RAGE [96].

NE is also an important component of NETs. Yazdani and colleagues found that NETs and mitochondrial biogenesis factors, including peroxisomes proliferator-activated receptor gamma coactivator 1-alpha (PGC-1α), mitochondrial transcription factor A (TFAM) and nuclear respiratory factor 1 (NRF-1), are increased in HCC and CRC tissues compared to their nontumor counterparts. NE directly alters metabolism and upregulates mitochondrial biogenesis in MC38 cells via the TLR4-PGC1-α pathway and thus promotes tumor growth [91].

In our ongoing study, we have illustrated that NETs can promote transcription factor IIB-related factor 1 (BRF1) expression in GC cells. BRF1 is known to affect the transcription and expression of RNA polymerase-3 related genes. In previous results, we found that BRF1 is a key molecule affecting the proliferation of GC [88].

Another relationship of note is that between NETs and tumor recurrence. Albrengues et al. induced sustained inflammation of the lung via tobacco smoke exposure or nasal instillation of LPS in Balb/c or nude mice that had been injected with MCF-7 breast cancer cells. They found an increase in NETosis and proliferation of awakened cancer cells in inflammatory lungs. NET DNA was prone to bind with the extracellular matrix (ECM) protein laminin. NETs awakened cancer cells and there was concomitant remodeling of laminin, which was sequentially cleaved by two NET-associated proteases, NE and MMP-9, through the activity of integrin α3β1 and focal adhesion kinase/ERK/myosin light chain kinase/yes-associated protein (FAK/ERK/MLCK/YAP) signaling [97].

### NETs promote tumor metastasis

**Fig. 3:**
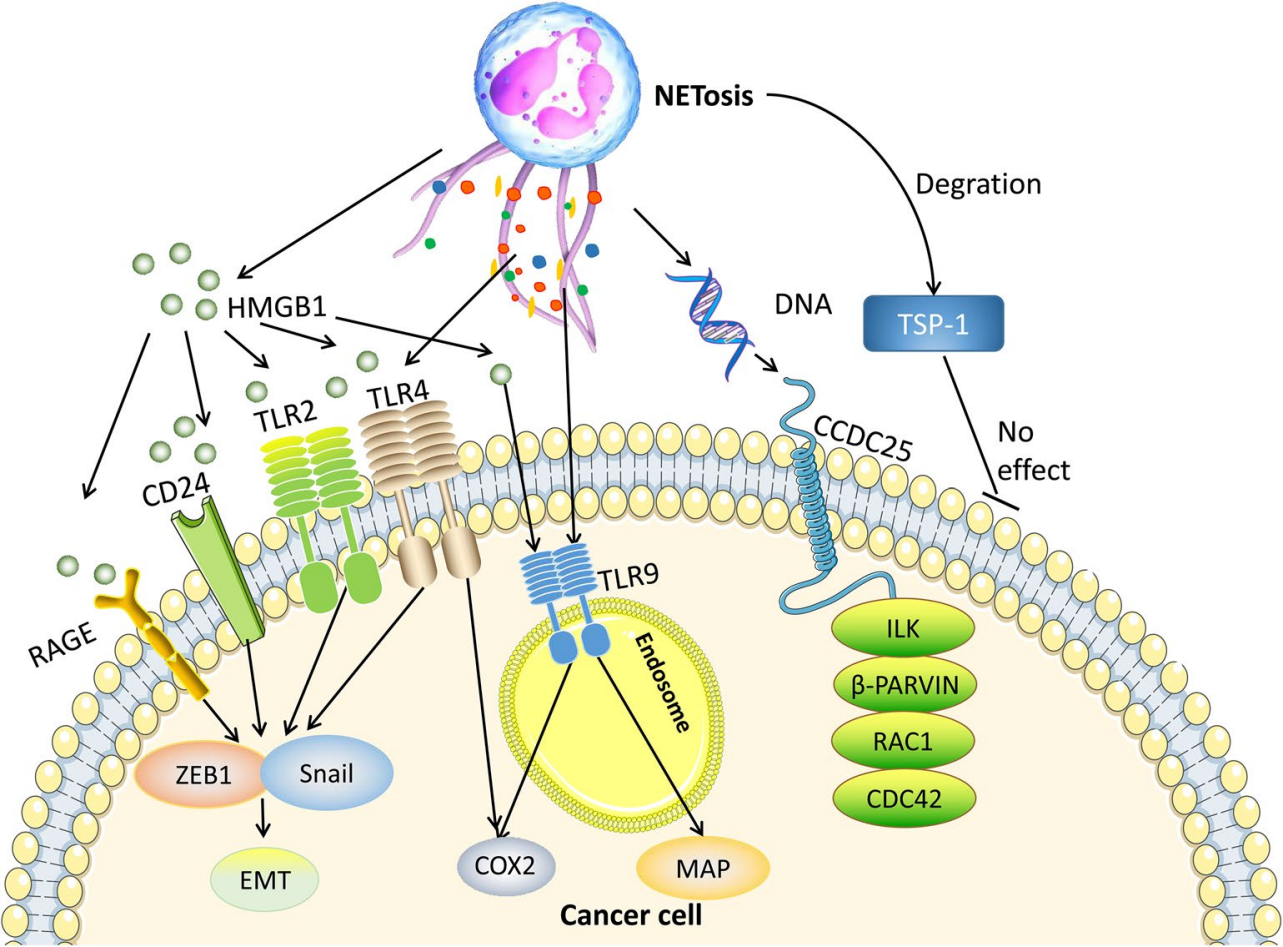
Molecular mechanisms of NETs in tumor metastasis. HMGB1, which is released by NETs, can facilitate tumor metastasis by the increased expression of EMT-associated genes, ZEB1 and Snail. In addition to EMT, NETs can also regulate expression of COX-2 and MAP to induce tumor migration. In human breast cancer cells, NET DNA binds to CCDC25 and induces an ILK-β-PARVIN-RAC1-CDC42 cascade, resulting in the metastasis of cancer cells. TSP-1 can be degraded by NETs to eliminate its inhibitory effect on tumor migration

With a deeper focus on the relationship between NETs and cancer, many studies have confirmed the mechanisms of NETs in tumor metastasis (Fig. 3). Park et al. [98] found that NETs promote migration and invasion in mouse 4T1 murine breast cancer cells and human BT-549 breast cancer cells. Similarly, an in vitro experiment showed that NETs promote migration in the human DLBCL cell lines SU-DHL2, SU-DHL4, and SU-DHL6 and the mouse DLBCL cell line A20 [38].

Many studies have reported the role of adhesion of NETs and cancer cells in tumor metastasis. Cools-Lartigue et al. [99] induced sepsis via cecal ligation and puncture and subsequently found that NETs promote cancer metastasis by trapping circulating tumor cells in circulation. This process is mediated by β1-integrin [100]. In an ovarian cancer model, NETs are located in the omentum, which is the premetastatic niche of ovarian cancer. NETs attach to ovarian cancer cells and promote metastasis [101].

Furthermore, another study found a novel pathway in breast cancer lung metastasis. NETs promote tumor metastasis by degrading thrombospondin-1 (TSP-1), a secreted extracellular matrix protein that inhibits tumor metastasis [26, 102].

An in vitro experiment showed that NET DNA significantly promotes the migration and adhesion of MDA-MB-231 human breast cancer cells. Proteins extracted from the cytoplasmic membrane of cancer cells and then inoculated with NET DNA were examined, and the transmembrane protein coiled-coil domain containing protein 25 (CCDC25) was found to function as a potential receptor for NETs to promote tumor metastasis. NET DNA binds to CCDC25 and induces an integrin linked kinase (ILK)-β-PARVIN-RAC1-CDC42 cascade, resulting in the metastasis of cancer cells [103].

HMGB1 played an important role in tumor metastasis [104, 105]. A study demonstrated that NETs can release HMGB1 and promote tumor metastasis [21]. Tohme et al. established an ischemia and reperfusion model to mimic surgical stress. They found that NETs induced by surgical stress promoted the development of gross metastases, which was mediated by HMGB1 from NETs through the TLR9-associated MAP kinase pathway in MC38 cells.

Endothelial to mesenchymal transition (EMT) is a key process by which cancer cells acquire migration ability [90]. Glomerular NETs have been confirmed to promote EMT in lupus nephritis patients and mouse models [11]. An in vitro experiment revealed that NETs can change the morphology of MCF-7 cells, accompanied by an increase in the transcription of EMT-related genes, such as zinc-finger E-box-binding homeobox 1 (ZEB1) and Snail. NETs promote migration ability in breast cancer through EMT [90]. This result is consistent with another study in GC [106]. It shows that process of EMT is significant for NETs induced tumor metastasis.

An interesting study assessed EMT, HMGB1 and NETs in cancer. NETs enhanced the migration and invasion of pancreatic cancer cells both in vivo and in vitro. Furthermore, the morphology of PANC-1 cells changed in the presence of NETs, and the transcription of genes involved in EMT, such as slug, ZEB1 and Snail, was upregulated. Immunofluorescence verified the colocalization of NETs and HMGB1. In this study, HMGB1 derived from NETs increased migration and invasion abilities through the EMT program [107].

In addition to the above pathways, the tumor microenvironment is also associated with tumor metastasis. The level of cyclooxygenase-2 (COX-2) is increased in MDA-MB-231 breast cancer cells that are treated with NETs. In addition, NETs induce a proinflammatory response [90]. An interesting experiment reported that the inflammatory response in the tumor microenvironment potentiates the metastatic potential of cancer via NETs. NETs promote metastasis of hepatocellular carcinoma by trapping HCC cells and promoting angiogenesis. In one study, RNA sequencing confirmed that a set of genes coding inflammatory mediators, for example, COX2, were upregulated. Related siRNAs were used to block the activity of TLR4/9, and in response, the NET-induced expression of COX2, which is downstream of TLR4/9, was obviously decreased [108]. These studies demonstrate that NETs promote potential metastasis of tumor via provoking tumorous inflammatory response.

### NETs promote coagulation and cancer-associated thrombosis

**Fig. 4:**
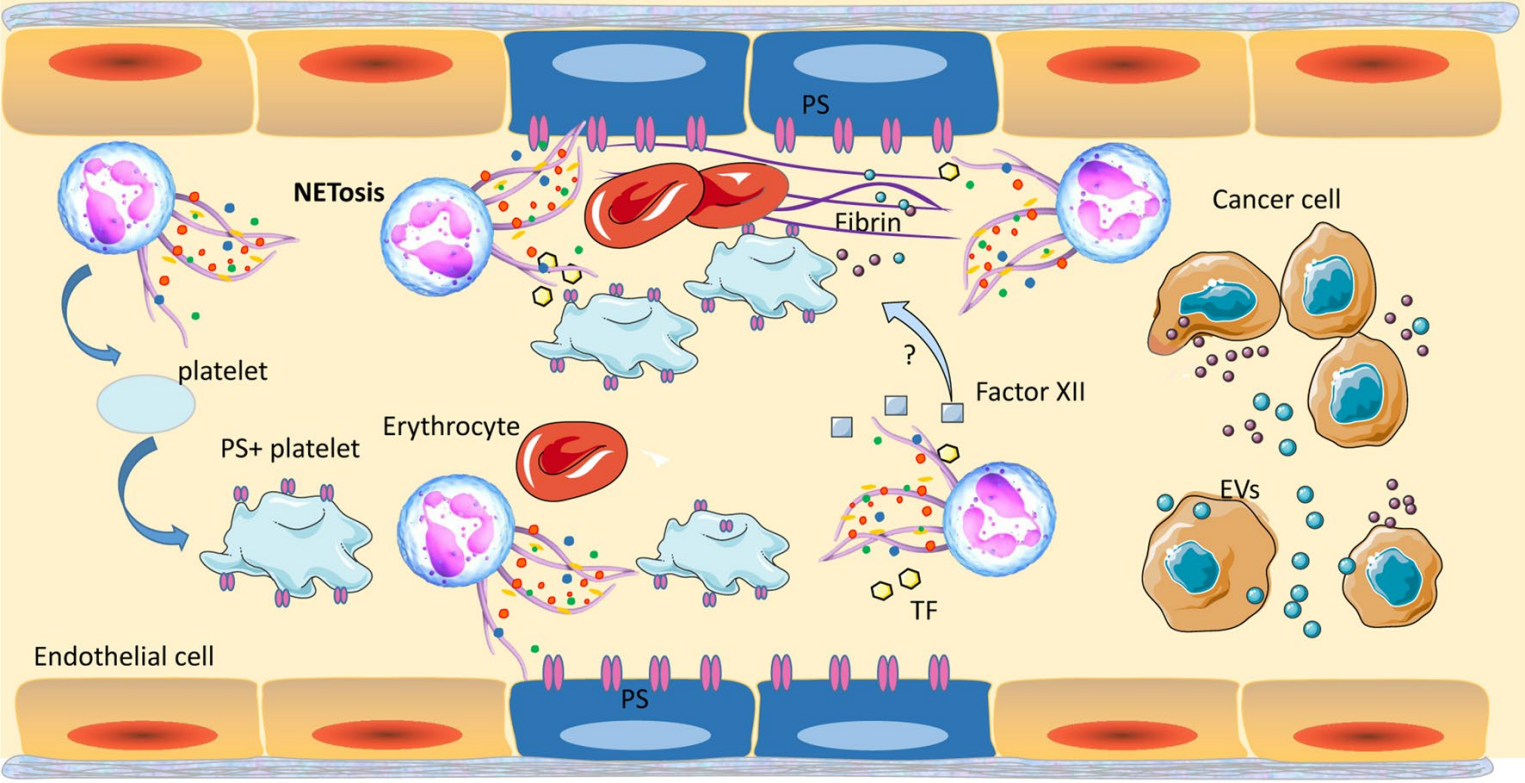
NETs promote coagulation and cancer-associated thrombosis. NETs provide a scaffold for platelets and tumor-derived EVs. Exposure of PS on platelets and endothelial cells that are induced by NETs promotes coagulation and cancer-associated thrombosis. EVs, which recruited by NETs, lead to a prothrombotic state. TF, which is derived from EVs or NETs, can promote cancer-associated thrombosis by initiating extrinsic coagulation. The factor XII, which can trigger the contact system, is associated with the level of NETs. However, the mechanisms by which NETs drive factor XII- and cancer-associated thrombosis need next studies

Cancer patients commonly have a hypercoagulable condition, and thrombosis has been identified as an important cause of death in cancer patients [27]. NETs were first found to promote thrombosis in an infected wound [109]. Subsequently, various studies confirmed that NETs attributed to atherosclerosis and thrombosis [110]. Recently, a role of NETs in coagulation and cancer-associated thrombosis was identified (Fig. 4). NETs induced by cancer cell could promote not only cancer progression but also hypercoagulability [111]. Spontaneous NETosis was found to be associated with thrombosis at late stages of cancer in mammary tumor-bearing mice [27]. In colorectal cancer patients, the activated partial thromboplastin time is significantly shorter and d-dimer levels are obviously higher than those in healthy subjects. Treatment with DNase I decreases coagulation time in control plasma treated with NETs [112]. Similarly, administration of DNase I significantly reduces the procoagulant role of NETs released by neutrophils derived from patients with GC [113]. NETs are crucial for the generation of venous and arterial thrombi in both cancer patients and tumor-bearing mice [40, 44, 114].

NETs have been shown to promote thrombin formation by providing a scaffold for platelets. Studies have shown that NETs trap platelets to promote platelet activation and thrombus formation [109, 115]. NETs derived from CRC patients promote the procoagulant activity of platelets and phosphatidylserine (PS) exposure on platelets. PS provides the specific catalytic surface needed for the coagulation cascade. Moreover, the study also revealed an increase in PS exposure on human umbilical vein endothelial cells (HUVECs) that had been stimulated with NETs from CRC. CRC-derived NETs induced shortened coagulation time and massive fibrin release from endothelial cells. NETs promoted the coagulant activity of endothelial cells mostly via PS [112]. Another study confirmed that NETs derived from pancreatic cancer convert HUVECs towards the procoagulant phenotype via PS [116]. All above results implies that exposure of PS induced by NETs on platelets and endothelial cells plays an important role in procoagulant activity in tumor.

NETs also serve as the backbone for tumor-derived extracellular vesicles (EVs) [44, 117]. Exosomes and microparticles (MPs) are two common types of EVs [44]. Exosomes are the products of the endolysosomal pathway and range in size from 30 to 150 nm [118]. NETs promote both venous and arterial thrombosis in a murine 4T1 mammary carcinoma model. Exosomes derived from 4T1 cells exhibit a procoagulant effect in a dose-dependent manner and tend to adhere to NETs in vitro. These results suggest that NETs might lead to a prothrombotic state in 4T1-bearing mice via recruitment of tumor-derived exosomes [44]. Moreover, another study found pancreatic tumor-derived MPs expressing tissue factor (TF) bound to NETs in vitro [117]. MPs are cell-derived membrane fragments that range in size from 0.1 to 1 μm [119]. In a deep vein thrombosis model generated by inferior vena cava stenosis, PANC02-derived MPs were infused into the blood of mice. As a result, the MPs adhered to NETs and accumulated at the site of pathological thrombosis, which promoted the formation of cancer-associated deep vein thrombosis in vivo [117].

TF is a transmembrane protein that functions as a receptor and activator of factor VII, subsequently initiating extrinsic coagulation [120, 121]. Some studies have confirmed that TF derived from MPs is correlated with coagulation activation and thrombosis in cancer [120, 122]. Furthermore, TF is also derived from neutrophils and released during the formation of NETs [123]. NETs have been shown to express functional TF [124, 125]. Another study confirmed that NETs can promote cancer-associated thrombosis by trapping tumor-derived MPs expressing TF in vitro [117]. In specimens of colonic adenocarcinoma and respective metastatic lymph nodes, TF colocalized with NETs [126]. In a model of murine pancreatic adenocarcinoma, coagulation was reduced via a decrease in the levels of TF, which was induced by abolishment of NETs [123]. It shows that TF modulated by NETs is vital for coagulation.

Factor XII can trigger the contact system, which initiates the intrinsic coagulation pathway [127]. In patients with HCC, the plasma levels of NETs and factor XIIa are elevated [128]. Similarly, markers of the contact system in plasma, including activated factor XIIa and high-molecular-weight kininogen, are significantly increased in acute leukemia samples compared with normal controls. The factor XIIa level is significantly correlated with the level of histone-DNA complexes, which seem to be a marker of NETs. Thus, contact system activation is associated with NET formation [129]. NETs have been shown to bind with factor XII via their negatively charged surface and subsequently activate intravascular thrombus formation [130]. However, the mechanisms by which NETs drive factor XII- and cancer-associated thrombosis need further investigation.

## The clinical value of NETs

In recent years, the value of NETs in cancer diagnosis, efficacy prediction and prognosis has been concerned. In our previous study, we discovered that NETs had novel diagnostic, therapeutic predictive, and prognostic value in GC patients [22]. Besides, elevated levels of NETs were associated with higher mortality in patients with cancer [131]. Recently, a study demonstrated that NETs were linked to a poor prognosis in patients with terminal cancer [132]. Thus, if we find the related way to inhibit the pathway of NETosis, it may control tumor invasion, evasion and metastasis, and ultimately improve the prognosis of cancer patients [133]. At present, NETs become a potential therapeutic target to inhibit cancer progression and metastasis [134, 135]. Some experiments in vivo have further proven this hypothesis. It has been confirmed that blocking NETosis via drugs against the components of NETs can effectively decrease ability of tumor growth and metastasis, such as DNase [21, 44], PAD4 inhibitors [97, 98, 136] and NE inhibitors [99]. Notably, a recombinant human DNase which named Pulmozyme was applied in a phase 1 trial in patients with head and neck cancer [134]. Danshen, the dried root of *Salvia miltiorrhiza*, which was traditional Chinese medicine, was found that it could exert antitumor effect via suppression of NETosis [137]. Moreover, inhibitors of CXCR1 and CXCR2, as the receptors of NETosis, were confirmed to block NETs [35, 36]. Meanwhile, the inhibitor of TLR9 which was a receptor of NETosis could retard tumor progression [38]. It provides a potential treatment about blocking key points of NETosis may control the progression of cancer effectively.

## Conclusions and future perspectives

Shortly after their discovery, NETs were recognized for their function in host defense against pathogens. Once NETs were reported in cancer, the relationship between tumors and NETs received more attention. In this review, the regulatory mechanism of NETs in cancer biological behavior is discussed in detail. Some stimuli that are exogenous or secreted from tumors, such as IL-8, G-CSF, PMA, LPS, HMGB1, and CTSC, have been shown to promote the formation of NETs in cancer. NETs can promote cancer cell proliferation and cancer progression via many kinds of pathways, including the TLR and RAGE pathways. Moreover, NETs potentiate metastasis by changing the adhesion of cancer cells, promoting EMT, and enhancing inflammation in the microenvironment. In addition, NETs can promote cancer-associated thrombosis by providing scaffolds for activated platelets and mediating coagulant molecules via EVs. In particular, NETs regulate tumor biological behaviors through interaction with tumor microenvironment, which is different from traditional molecule targets. Because, NETs is a complex structure in tumor microenvironment, not a single molecule. It reflects the new function of infiltrated neutrophils in the tumor microenvironment. Analysis of the molecular mechanisms underlying NET formation has revealed that some key receptors and signaling pathways participate in both NETosis and tumor biological behaviors.

Interestingly, we found that NETs could promote tumor progression in a variety of tumor types, such as GC [22], head and neck cancer [23], DLBCL [38] and breast cancer [103]. On the other hand, the presence of HCC did not further increase the levels of NETs as compared to patients with cirrhosis only [138]. However, the presence of NETs accelerated transition of NASH to HCC [92]. It indicates that heterogenous presence of NETs in cancer progression may attribute to the complex causes of different cancers. In general, we still suggest that tumor-associated inflammation represented by NETs may be one of the common mechanisms to trigger cancer progression.

Given their crucial roles in cancer, NETs are possible therapeutic targets in cancer patients. Blocking NETosis through drugs against the components of NETs or the receptors of NETosis can effectively decrease ability of tumor growth and metastasis. Similarly, in recent years, immunotherapy by targeting T lymphocytes in the tumor microenvironment has changed the current state of medical antitumor therapy. Accordingly, we hope that NETs will become possible therapeutic targets for inhibiting proliferation, metastasis and thrombosis in cancer patients in the future.

## Data Availability

Not applicable.

## Abbreviations

NETs: Neutrophil extracellular traps
NE: Neutrophil elastase
MPO: Myeloperoxidase
MMP-9: Matrix metalloproteinase 9
CG: Cathepsin G
PR3: Proteinase 3
ROS: Reactive oxygen species
PAD4: Peptidylarginine deiminase 4
LPS: Lipopolysaccharide
PMA: Phorbol 12-myristate 13-acetate
HMGB1: High mobility group 1
CTSC: Cathepsin C
G-CSF: Granulocyte colony-stimulating factor
IL-8: Interleukin-8
CXCL8: C-X-C motif chemokine ligand 8
NSCLC: Non-small cell lung cancer
CXCR1: C-X-C motif chemokine receptor 1
CXCR2: C-X-C motif chemokine receptor 2
DLBCL: Diffuse large B cell lymphoma
ERK: Extracellular-signal-regulated kinase
PI3K: Phosphoinositide 3-kinase
AKT: Serine/threonine-protein kinase
GC: Gastric cancer
PKC: Protein kinase C
MEK: MAP kinase ERK kinase
MAPK: Mitogen-activated protein kinase
Syk: Spleen associated tyrosine kinase
JNK: C-jun N-terminal kinase
TLR4: Toll-like receptor 4
IRF-1: Interferon regulatory factor 1
RAGE: Receptor for advanced glycation end products
TNF: Tumor necrosis factor
RIPK1: Receptor-interacting-protein kinase-1
IL-1β: Interleukin-1β
NASH: Nonalcoholic steatohepatitis
HCC: Hepatocellular carcinoma
STAT3: Signal transducer and activator of transcription 3
CRC: Colorectal cancer
PGC-1α: Peroxisomes proliferator-activated receptor gamma coactivator 1-alpha
TFAM: Mitochondrial transcription factor A
NRF-1: Nuclear respiratory factor 1
ECM: Extracellular matrix
FAK: Focal adhesion kinase
MLCK: Myosin light chain kinase
YAP: Yes-associated protein
TSP-1: Thrombospondin-1
CCDC25: Coiled-coil domain containing protein 25
ILK: Integrin linked kinase
EMT: Endothelial to mesenchymal transition
ZEB1: Zinc-finger E-box-binding homeobox 1
COX-2: Cyclooxygenase-2
PS: Phosphatidylserine
HUVECs: Human umbilical vein endothelial cells
EVs: Extracellular vesicles
MPs: Microparticles
TF: Tissue factor
